# Augmented Renal Clearance in Critical Illness: An Important Consideration in Drug Dosing

**DOI:** 10.3390/pharmaceutics9030036

**Published:** 2017-09-16

**Authors:** Sherif Hanafy Mahmoud, Chen Shen

**Affiliations:** Faculty of Pharmacy and Pharmaceutical Sciences, University of Alberta, Edmonton, AB T6G 1C9, Canada; cshen2@ualberta.ca

**Keywords:** augmented renal clearance, enhanced renal function, critically ill

## Abstract

Augmented renal clearance (ARC) is a manifestation of enhanced renal function seen in critically ill patients. The use of regular unadjusted doses of renally eliminated drugs in patients with ARC might lead to therapy failure. The purpose of this scoping review was to provide and up-to-date summary of the available evidence pertaining to the phenomenon of ARC. A literature search of databases of available evidence in humans, with no language restriction, was conducted. Databases searched were MEDLINE (1946 to April 2017), EMBASE (1974 to April 2017) and the Cochrane Library (1999 to April 2017). A total of 57 records were included in the present review: 39 observational studies (25 prospective, 14 retrospective), 6 case reports/series and 12 conference abstracts. ARC has been reported to range from 14 to 80%. ARC is currently defined as an increased creatinine clearance of greater than 130 mL/min/1.73 m^2^ best measured by 8–24 h urine collection. Patients exhibiting ARC tend to be younger (<50 years old), of male gender, had a recent history of trauma, and had lower critical illness severity scores. Numerous studies have reported antimicrobials treatment failures when using standard dosing regimens in patients with ARC. In conclusion, ARC is an important phenomenon that might have significant impact on outcome in critically ill patients. Identifying patients at risk, using higher doses of renally eliminated drugs or use of non-renally eliminated alternatives might need to be considered in ICU patients with ARC. More research is needed to solidify dosing recommendations of various drugs in patients with ARC.

## 1. Introduction

Studying the influence of renal dysfunction on the pharmacokinetics of drugs is an important consideration in drug development. In addition, clinicians are vigilant in adjusting the doses of renally eliminated drugs in patients with various degrees of renal impairment to avoid potential toxicities. On the other hand, little attention is given if patients exhibit an augmented renal clearance (ARC). Augmented renal clearance (ARC) is a manifestation of enhanced renal function seen in critically ill patients [1,2]. It is currently defined as an increased creatinine clearance of greater than 130 mL/min/1.73 m^2^. ARC is a clinical phenomenon rapidly gaining recognition in the world of critical care. Although ARC may have existed long before our current recognition, it wasn’t until the early 2010’s, that the research group led by Andrew Udy put forth the concept of ARC as an independently existing medical phenomenon [3]. The increased renal clearance of endogenous by-products, various chemicals, toxins, and most importantly medications during ARC manifestation may have a significant impact on patient outcome. The use of regular unadjusted doses of renally eliminated drugs in patients with ARC might lead to therapy failure and worse patient outcome. The purpose of this scoping review was to provide and up-to-date summary of the available evidence pertaining to the phenomenon of ARC including epidemiology, risk factors and pathophysiology of ARC, pharmacokinetic changes of drugs in patients with ARC and suggested assessment and management approach of patients exhibiting ARC.

## 2. Methods

A literature search of databases of available evidence pertaining to augmented renal clearance (ARC) in humans, with no language restriction, was conducted. Databases searched were MEDLINE (1946 to 12 April 2017), EMBASE (1974 to 12 April 2017) and the Cochrane Library (1999 to 12 April 2017). To ensure that we captured all studies involving augmented renal clearance, we used the following keywords: “augmented renal clearance”, “enchanc * renal clear *”, “increase * renal clearance”, “augmented kidney clearance”, “enhance * kidney function *”, “ren * ultrafiltrat *”, “enhance * creatinine clear *”, “increase * kidney function *”, and “increase * creatinine clear *”. Title and abstract screening were then conducted to identify duplicate studies and studies that were clearly not pertaining to the topic for exclusion. If any doubt arose regarding whether a study was related to ARC, the study was included for full text review. Studies on renal dysfunction (e.g., acute kidney injury, chronic kidney disease, renal dysfunction, etc.) or other clinical phenomena that would alter drug elimination (e.g., cystic fibrosis) were excluded. Non-human studies, non-English studies that could not be easily translated into English using an online translator tool, commentaries, opinion articles, editorials and review articles were excluded. Both authors independently conducted the processes of screening. Then, the full texts of the selected articles were assessed for inclusion in our review. Lastly, a manual search for additional relevant studies was performed by analyzing the reference lists of the selected studies. In case of any discrepancies between the reviewers, further discussion was done to reach a consensus. Data extraction from studies was confirmed by both authors.

## 3. Results and Discussion

**Figure 1:**
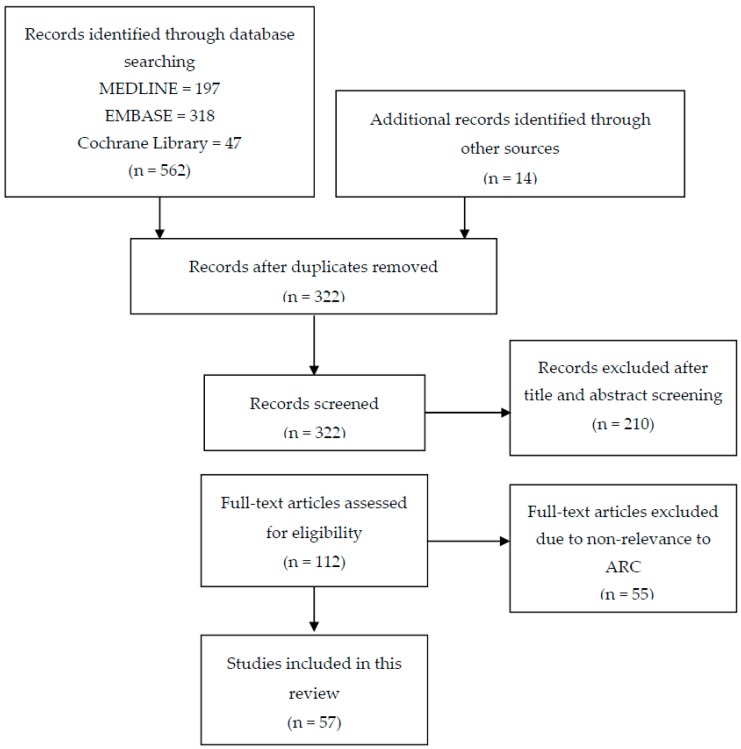
Flow diagram of the literature search for studies addressing augmented renal clearance (ARC).

As depicted in Figure 1, databases search resulted in 562 records. After duplicate removal and addition of records from other sources, 322 records remained. After title, abstract and full text screening, a total of 57 records were included in the present review: 39 observational studies (25 prospective, 14 retrospective), 6 case reports/case series and 12 conference abstracts. Because the main body of evidence was derived from observational studies, caution should be exercised while interpreting the results of the included records especially case reports. Table 1 summarizes the studies included in this review.

### 3.1. ARC Definition and Prevalence

Augmented renal clearance (ARC), also reported as glomerular hyperfiltration or enhanced renal clearance, is an increase in kidney function that results in enhanced clearance of drugs with potential for therapy failure. ARC has been defined using creatinine clearance (CrCl). However, the definition of ARC in relation to CrCl cutoff has varied among studies, thus impeding accurate identification of ARC prevalence among intensive care unit (ICU) patients. Although, many research groups have defined ARC as patients with creatinine clearance > 130 mL/min/1.73 m^2^ (Table 1), other creatinine clearance cutoffs have been suggested including ARC cutoff of CrCl > 120 mL/min/m^2^ [26,38,41,55,58], and >160 mL/min/1.73 m^2^ [11]. The definition of ARC was further complicated with the duration at which CrCl above the suggested cutoff. The majority of the studies have considered one occurrence of CrCl above cutoff is sufficient to acknowledge the presence of ARC. However, both Baptista et al. [24] and DeWaele et al. [15] elected to use the definition of ARC as CrCl > 130 mL/min/1.73 m^2^ for more than half of the CrCl measures during a minimum of 72 h of ICU stay. This acknowledges the concern if the current definition of CrCl cutoff (without timeframe specification) truly captures the clinical implication (i.e., the point where changes must be made for renally cleared medications) to patient care. Furthermore, it is not clear if additional cutoffs beyond CrCl of 130 mL/min/1.73 m^2^ are needed to stage ARC parallel the categories used to describe renal dysfunction i.e., mild, moderate and severe ARC. However, despite the various definitions observed, based on the large number of studies using the definition CrCl cutoffs of >130 mL/min/1.73 m^2^, as well as associated clinical implications at this cut off point, we recommend a unified definition of ARC using CrCl > 130 mL/min/1.73 m^2^ as the clinical cut-off in the adult population.

ARC has been reported to range from 14 to 80% (Table 1), suggesting that ARC is a commonly occurring clinical phenomenon. However, these studies may perhaps over- or under-estimate the true prevalence of ARC due to few reasons. First, the most common practice setting where ARC was identified is within the setting of the ICU. Since rigorous patient monitoring is a common practice within the ICU, including daily measures of renal function, it is much easier to identify ARC. In addition, critically ill are exposed to factors that may increase the likelihood of ARC occurrence, such as the use of intravenous fluids, vasopressors and inotropes [60,61]. Second, as discussed above, variations in ARC definitions might impede the true prevalence of ARC. Third, ARC prevalence needs to be interpreted in the context of the study patient selection criteria. For example, exclusion of patients with renal dysfunction will result in higher percentage of patients with ARC and vice versa.

The true onset and duration of ARC in critically ill is not known. In studies that assessed renal function more than one occasion throughout the ICU stay, it appears that the onset of ARC coincides with an acute insult to the body. In a prospective observational study of patients admitted to ICU, Udy et al. have reported that 65% of patients, identified to have ARC, had at least 1 occasion of measured ARC during the first 7 days of admission with 38% of those had ARC on the first day of ICU admission [29]. Occurrence of ARC on day 1 of ICU admission significantly predicted sustained CrCl elevation over the first seven days of the ICU stay (*p* = 0.019). Similarly, Huttner et al. have reported that 64% of patients had ARC at study enrollment [17]. Duration of ARC has varied among studies owing to difference in monitoring frequency and duration. While many studies have reported persistence of ARC for weeks [17,43,45,46,58], fewer patients exhibited transient ARC lasting for no more than 1 day [31]. Given the current unpredictability of ARC duration, continuous monitoring of patient’s renal function is warranted as alteration in drug dosing might be required.

### 3.2. Pathophysiology

Our current understanding of the ARC pathophysiology remains limited. It has been reported that ARC is associated with increased glomerular filtration, renal tubular secretion of anions, and renal tubular reabsorption using various exogenous markers, suggesting that ARC affects many components of the nephron physiology [28]. It has been suggested that ARC is a hyperdynamic response to insults to the body. In the early study conducted by Ljungberg and Nilsson-Ehle, acute infection has been observed to be associated with enhanced renal clearance [62]. This has been attributed to changes in vascular permeability and increased renal blood flow secondary to elevated body temperature. Similarly, the effect of temperature changes on renal function has been reported in patients exposed to induced hypothermia. In a retrospective study comparing vancomycin pharmacokinetic in patients with controlled normothermia (median temperature = 37.2 °C) to patients with induced hypothermia (median temperature = 34 °C), Morbitzer et al. have demonstrated that vancomycin clearance was higher in controlled normothermic patients [18].

In addition to altered body temperature, insult to the brain could lead to ARC. Dias et al. have identified a possible link between the brain and the kidneys in their retrospective analysis of 18 severe traumatic brain injury (TBI) patients managed with intracranial pressure monitoring in the neurocritical care setting [16]. Analyzing cerebrovascular pressure reactivity index (PRx, a correlation index between intracranial pressure and arterial blood pressure that reflects the capability cerebral arteries to react to changes in blood pressure and is a key element of cerebral autoregulation) in ARC-manifesting patients showed a strong negative correlation (*r* = −0.81, *p* < 0.001) between PRx and creatinine clearance. This correlation suggests that reduction in cerebral autoregulation (i.e., after a TBI) is associated with an increase in creatinine clearance, adding an evidence to the theory that the central autoregulation plays a significant role in the manifestation of ARC. In another study of 11 TBI patients by Udy et al., atrial natriuretic peptide (ANP) levels have been found to be elevated in TBI patients suggesting that ANP following brain may play a role in enhancing glomerular filtration through increased natriuresis and diuresis [7]. The results of the correlation between ANP and CrCl, however, did not reach statistical significance.

Sime et al. have proposed a model of hyperdynamic state to bring forth a pathophysiology model to the occurrence of ARC [63]. It has been suggested that a number of factors consequential to critical illness combine to produce ARC. Systemic inflammatory response syndrome (SIRS) associated with critical illness results in the increase of inflammatory mediators. These mediators produce decrease in vascular resistance in the peripheries and increase in cardiac output. These two responses combine to produce a hyperdynamic state within the body system, resulting in increased renal blood flow, followed by glomerular hyperfiltration that manifest itself as ARC. Additionally, common to the ICU setting, critically ill patients are often subjected to fluid therapy and treatment with vasoactive drugs and inotropes thereby further increasing cardiac output that would more likely to contribute to the already-increased hyperdynamic state. Furthermore, critical illness also may have direct effect on the kidneys, further enhancing renal clearance in ARC. Although this model suggested by Sime et al. provides a logical explanation to the pathophysiology leading to ARC, the exact mechanism to which ARC, as a sequelae of physiological insult, remains uncertain.

### 3.3. ARC Risk Factors

Various studies (Table 1) have shown that patients exhibiting ARC tend to be younger (<50 years old), of male gender, had a recent history of trauma, and had lower critical illness severity scores such as sequential organ failure assessment score (SOFA) [35], Simplified Acute Physiology Score (SAPS) II [41] or Acute Physiology and Chronic Health Evaluation (APACHE II) [35,51]. Young age appears to be the only risk factor consistently recognized by various epidemiology studies to be able to reliably predict ARC (Table 1). In addition, Hirai et al. have identified febrile neutropenia to be an independent risk factor of ARC the pediatric cancer population [11].

Recognizing the need for a clinical prediction tool to identify patients at risk for manifesting ARC, Baptista et al. [53] have conducted a retrospective study in 447 patients admitted to an ICU at a tertiary hospital over a 1-year period, and assessed patient characteristics on its predictability of ARC occurrence. Urinary creatinine > 45 mg/mL and age < 65 years have been identified as best predictors of ARC (sensitivity 60%, specificity = 88%); specificity increased to 95% by adding BUN < 7 mmol/L. Furthermore, Udy et al. [35]. developed an ARC scoring system based on the risk of factors of age < 50 years old, presence of trauma, and SOFA score ≤ 4 (Table 2). This predictive tool was later validated by Akers et al., demonstrating a sensitivity of 100% and specificity of 71% for detecting patients with ARC, based on confirmation data from altered piperacillin/tazobactam pharmacokinetics in ICU patients [23]. Because of the impracticality need to complete a SOFA score in ARC scoring system, Barletta et al. have developed the augmented renal clearance in trauma intensive Care (ARCTIC) scoring system (Table 2) [4]. The ARCTIC scoring system employed the patient factors: serum creatinine, sex and age to identify those with high ARC risk (ARCTIC score > 6). The ARCTIC scoring system produced a sensitivity of 84% and specificity of 68%. Given the need for early recognition of ARC in the ICU setting, the use of the ARC or ARCTIC predictive tools allow for identification of at risk patients, and help direct clinicians to take appropriate interventions (e.g., obtain a measured creatinine clearance, employ more aggressive antibiotic dosing regimen/strategies, etc.).

### 3.4. Creatinine Clearance: Estimation Methods and ARC

Although risk factors and predictive models offer a method to screen for those at an increased risk for ARC, the actual identification of ARC requires accurate glomerular filtration rate (GFR) determination. While determination of inulin clearance is regarded as the gold standard for measuring GFR because CrCl might overestimate kidney function, affected by muscle mass and physical activity, urinary measurement of creatinine clearance (from 8 to 24 h of urine collection) is currently the most common measurement of renal function in the clinical setting [64]. That’s because inulin clearance determination is labor intensive and requires administration of an exogenous substance. Because routine measurement of creatinine clearance is impractical, mathematical estimates of creatinine clearance based on population parameters are often employed to allow for prompt determination. Commonly used mathematical estimates of creatinine clearance/glomerular filtration rate (GFR) include the Cockcroft Gault equation (CG), the Modification of Diet in Renal Diseases (MDRD) formulae, and the Chronic Kidney Disease-Epidemiology (CKD-EPI) equation. These equations all have been validated in various populations, and their respective merits and deficiencies have been described elsewhere [65]. Measured creatinine clearance has been reported in 59% of the included studies. On the other hand creatinine clearance has been estimated only or not reported in 25 and 16% of the included studies, respectively (Table 1). In studies assessing the accuracy of mathematical estimates of creatinine clearance in the ARC population, all the mathematical estimations of creatinine clearance have been found to underestimate the actual measured creatinine clearance in patients with ARC [8,19,21,22,24,36,38,40,50,57].

Comparison of the various mathematical estimates suggest the Cockcroft Gault (CG) formula may be the best method of creatinine clearance estimation in the ARC population. To illustrate, Udy et al. have assessed the accuracy of the CKD-EPI estimation of creatinine clearance in comparison to 8-h measured creatinine clearance in 110 ICU patients [36]. Around 42% of the patients identified by the CKD-EPI equation to have creatinine clearance within the range of 60–119 mL/min/1.73 m^2^ exhibited ARC (measured CrCl > 130). In a study conducted by Baptista et al., the CG and MDRD (4- and 6-variable) estimations of creatinine clearance were compared to measured creatinine clearance using 8-h or 24-h urine sampling [40]. In patients exhibiting ARC, CG estimate was able to detect 62% of the cohort exhibiting ARC, while the MDRD estimations demonstrated lower sensitivity, with the 4-variable MDRD formula detecting 47% of the cohort while the 6-variable MDRD formula was only able to detect 27% of the cohort exhibiting ARC. Similarly, Barletta et al. have found that measured creatinine clearance were significantly higher (*p* < 0.001) than all estimates of creatinine clearance (CG, 4-variable MDRD, and CKD-EPI equation) with the CG method demonstrating the lowest bias [8]. However the CG method may only be used as a screening tool for ARC. Even if a CG estimate show an estimated creatinine clearance within the normal reference range, there is a still a high likelihood that ARC can be present in a patient. Therefore, in the setting of ICU, it would be prudent to assess a patient’s measured creatinine clearance at least once, to determine whether a patient is truly experiencing ARC, and to also gauge the level of bias in the estimated creatinine clearance for that patient. Finally, we would like to mention that although serum creatinine used for determination of creatinine clearance is a reliable marker within the general population, consideration must be made when applying this measurement in patients with lower muscle mass, immobility, children or other conditions in which muscle mass are altered. Due to the reduced production of creatinine in these patients, falsely low measures of serum creatinine may inaccurately identify ARC in those populations.

### 3.5. Drug Therapy in ARC Population

#### 3.5.1. Pharmacokinetic Changes in ARC

In the ICU setting, pharmacokinetic changes to drug therapy in the presence of ARC may have drastic implications on patient outcome. This is especially important to drugs that are renally cleared known to exhibit direct correlation between their renal clearance and creatinine clearance such as aminoglycoside antimicrobials, vancomycin and levetiracetam. Enhanced drug clearance will lead to shorter drug half-life (*t*_½_), lower maximum drug concertation (*C*_max_) and lower area under the concentration curve (AUC) which could have direct implication on drugs’ pharmacodynamic effects leading to therapy failure. This particularly important for antimicrobials that are time-dependent killers (efficacy depends on the duration of the drug concentration or AUC above the minimum inhibitory concentration (MIC) of the pathogen T > MIC) and concentration-dependent killers (efficacy depends on the Cmax of the drug relative to the MIC of the pathogen). Currently, antimicrobial monograms and various dosing guidelines have not acknowledged the need for alterations to drug dosing regimen in the ARC population. In a survey of ICU physicians in England clarifying their attitudes regarding antibiotic prescribing and assessment of renal function in septic patients, only 15% responded that they would consider modifying the dosage regimen of beta-lactams and vancomycin antimicrobials in patients with ARC [52]. This highlights the need for dosing guidance in patients with ARC.

#### 3.5.2. Vancomycin

Vancomycin, a glycopeptide antibiotic, is one of the antimicrobials of choice for the treatment of serious, life-threatening infections by Gram-positive bacteria. It is a hydrophilic drug that is 80–90% excreted unchanged by the kidneys and its clearance is highly dependent on renal function. Previous pharmacokinetic and pharmacodynamic studies have established that steady state vancomycin trough level is a good surrogate of AUC:MIC which is in turn correlated with treatment success [66].

Currently, there is a growing body of evidence suggesting inadequate therapeutic vancomycin trough levels attained using conventional dosing in patients exhibiting ARC [9,25,26,32,43,45,50,55]. For example, Campassi et al. have reported that 100% of patients with ARC did not have vancomycin trough levels within the target 15–25 mg/L 3 days following vancomycin initiation despite getting high vancomycin doses [26]. Currently, studies on the clinical outcome of subtherapeutic serum concentration of vancomycin in ARC patients are scarce. However, it has been reported that in patients who did not reach therapeutic trough target 15–25 mg/L 48 h post treatment initiation, in-hospital mortality was significantly higher than those who have attained therapeutic target trough (OR = 2.1, *p* = 0.003) [20].

Various vancomycin dosing regimens have been suggested and tested in patients with ARC. In a study by Vermis et al., vancomycin therapeutic drug monitoring (TDM) has been implemented and vancomycin dose adjustment was based on vancomycin trough level 5 days post vancomycin initiation [55]. The average doses successful to achieve trough levels within target were 42 and 33 mg/kg/day in patients with ARC and without ARC, respectively. The authors proposed a loading dose of 25 mg/kg loading dose followed by 40 mg/kg/day for those with CrCl > 130 mL/min. Similarly, Minkute et al. have reported the need for vancomycin doses up to 44 mg/kg/day to achieve trough levels within target [32]. Baptista et al. [25] have suggested a vancomycin dosing strategy using 8 h measured creatinine clearance to achieve a target trough of 25 mg/L. The nomogram suggested has been validated in a second group of patients in the same study and has been found to produce 84% target attainment in patients with ARC.

#### 3.5.3. Beta-Lactam Antimicrobials

Beta-lactams antibiotics are primarily renally eliminated thereby are affected by presence of ARC. Unlike vancomycin or aminoglycosides, therapeutic drug monitoring is not common for beta-lactam antibiotics. Clinicians generally prescribe guideline-recommended regimens without the need to conduct therapeutic drug monitoring. Although following the patients' clinical status after prescribing a beta-lactam is appropriate and acceptable in the general population, dismissal of beta-lactam concentration in the ARC population, where very little evidence exists and no dosing guidelines have been recommended, poses a substantial threat to treatment success and patient outcome. Numerous studies have reported beta-lactam treatment failures (based on sub-therapeutic serum level attainment) when using standard beta-lactam dosing regimens in patients with ARC (discussed below and in Table 1).

##### Carbapenems

Carbapenems (such as meropenem, imipenem, doripenem and ertapenem) are a family of broad spectrum beta-lactams used for the treatment of multi-drug resistant bacteria. They exhibit time-dependent antibacterial activity and their activity can be best illustrated by the T > MIC pharmacodynamic model [67]. Various studies have reported poor target achievement in ARC patients using conventional regimens. Binder et al. have reported that standard meropenem doses (500–1000 mg every 8 or 12 h) in ICU patients with estimated creatinine >60 mL/min result in lower meropenem AUC secondary to increased clearance suggesting the need for alternate regimen in this population [68]. Similarly, Drust et al. have reported that almost two-thirds of the ICU patents with CrCl > 120 mL/min required higher doses (up to 8 g/day) of meropenem than current recommended therapy to achieve effective plasma meropenem concentrations [59]. In addition, extended infusion have also been suggested for appropriate target T > MIC target attainment and treatment success. Carlier et al. have assessed the efficacy of whether meropenem extended infusion (1 g over 30 min, followed by a maintenance dose of meropenem 1 g infused over a period of 3 h every 8 h) would be a suitable alternative strategy for meropenem dosing in ARC patients [30]. Extended infusion did not improve meropenem plasma concentration by the end of the study, with lower percentage of ARC patients (61%) achieving T > MIC therapeutic target in comparison to non-ARC patients (94%, *p* < 0.001). In addition to meropenem, doripenem pharmacokinetics have been studied in critically ill patients with ARC [33]. Similarly, higher doses of doripenem have been suggested in patients with ARC.

##### Piperacillin/Tazobactam

Piperacillin/tazobactam is an extended spectrum beta-lactam antibiotic indicated for the treatment of severe multi-drug resistant infections. Like all beta-lactam antibiotics, piperacillin/tazobactam exhibits time-dependent bacterial eradication (T > MIC). Piperacillin/tazobactam is eliminated renally, and dose adjustment of piperacillin/tazobactam has been described for renally impaired population. There are many reports of subtherapeutic target attainment using conventional piperacillin/tazobactam dosing in ICU patients with ARC. Huttner et al. have assessed target attainment of various beta-lactams in the ICU population (ARC observed in 64% of the study cohort) [17]. Subtherapeutic serum concentrations have been reported with intravenous doses of piperacillin/tazobactam 4.5 g every eight hours in 61% of the treated patients. In addition, undetectable piperacillin/tazobactam concentrations were seen in 7% of the patients. To address the issue of subtherapeutic target attainment of piperacillin/tazobactam especially in ICU patients with ARC, various dosage regimens have been tested and suggested [6,23]. A Monte-Carlo simulation conducted by Akers et al. have suggested that doses above the FDA approved doses (up to 36 g per day) might be needed to achieve high probability of target achievement [23]. Unfortunately, the study by Akers et al. was not aimed to assess any of the dosing regimens modelled in their study and these proposed regimens still require validation.

##### Other Beta-Lactam Antimicrobials

Aside from few other studies [14,48], the evidence of the other members of the beta-lactams is scarce. However, as suggested by the body of evidence on meropenem and piperacillin/tazobactam, it is likely that all beta-lactams that are eliminated renally might be affected by the ARC phenomenon and require further research.

#### 3.5.4. Other Medications

Aminoglycosides are mainly renally eliminated with predictable efficacy based on serum concentrations. Goboova et al. have conducted a retrospective analysis of 204 patients receiving gentamicin, in which 14% of the patients exhibited ARC [49]. The patients in the study were initially treated with conventional gentamicin regimen. Analysis of gentamicin peak concentration at steady state identified 93% of the ARC patients had subtherapeutic steady state concentrations which required dose escalation to attain target peak levels. This highlights the value of therapeutic drug monitoring in the setting of ARC.

Fluoroquinolones are another family of antibiotics affected by altering renal clearance. Due to predictability of current dosing in patients normal or impaired renal function, TDM is not necessary. However, TDM could be of value when using fluoroquinolones in patients with ARC and higher doses may be required. For example, using Monte Carlo simulation, levofloxacin doses of 1 g every 24 h have been suggested for infections caused by *S. pneumoniae*, *P. aeruginosa*, and *S. aureus* in patients with CrCl > 130 mL/min (conventional dosing 0.5–0.75 g every 24 h) [13].

In addition to antimicrobials, other agents have been tested such as levetiracetam and enoxaparin. Levetiracetam is a broad spectrum antiepileptic drug (AED) that has a more favorable side effect profile compared to older AEDs. It displays linear elimination kinetics; therefore dose changes produce relatively predictable changes in serum concentrations. However, it is renally eliminated and its clearance is directly proportional to CrCl and thus could be affected in patients with ARC. There are few reports discussing the enhanced elimination of levetiracetam in TBI and SAH patients with ARC [42,45,54]. Accordingly, Higher initial levetiracetam doses (1 g IV every 8 h) have been suggested in patients with high risk for ARC [42,54]. Enoxaparin, a low molecular weight heparin, also has been reported to be affected by presence of ARC suggested the need for more rigorous monitoring of anti-factor Xa activity in patients with ARC [5].

## 4. Conclusions

**Figure 2:**
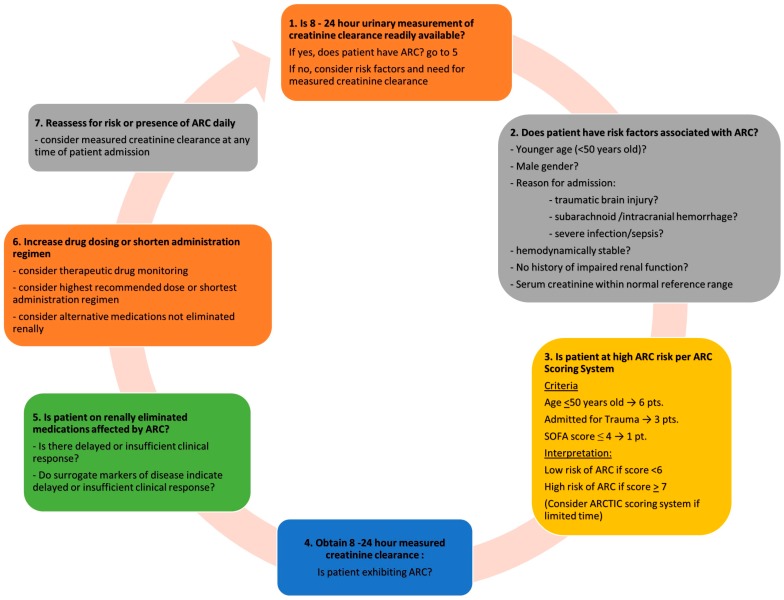
Assessment of ICU patients for augmented renal clearance (ARC).

Augmented renal clearance (ARC) is a manifestation of enhanced renal function seen in critically ill patients. The current evidence presented in this review identified the significance of this phenomenon and the need for higher doses of renally eliminated drugs in patients with ARC. More research is needed to solidify dosing recommendations of various drugs in patients with ARC. However, based on the current evidence few recommendations could be put forward to guide clinicians in managing patients presenting with ARC (Figure 2).

The recognition of ARC risk factors allows clinicians to screen for at-risk patients. In the ICU setting, the ARC-scoring tool (Table 2) could be used to identify patients suitable for further investigation. If time does not permit the implementation of the ARC-scoring tool, the less time-consuming ARCTIC scoring system (Table 2) could still be considered. Other means of identifying at-risk patients include assessment of patient serum creatinine, estimated creatinine clearance, or delayed clinical response to medication interventions. Upon identification of at-risk patients, a measured 8–24 h creatinine clearance study should be undertaken. Evidence has shown that estimated creatinine clearance carry significant risk of underestimating the renal function of ARC patients in various settings. Therefore, at minimum, determination of a patient’s creatinine clearance may be conducted by employing an 8-h urinary measurement, which will aid in the diagnosis of ARC and later-on be used for dosage adjustment of renally cleared medications. We also recommend continued daily monitoring of serum creatinine for those diagnosed with ARC. Due to the unpredictability of ARC manifestation (could be a transient 1-day occurrence or maintained for weeks), we can only determine the manifestation of ARC in an individual through continued measurement of renal function.

Despite many studies seem to suggest that ARC tends to manifest itself during initial ICU admission, any patients, including those with a recent history of acute kidney injury, could exhibit ARC at any time of their hospital admission. For those patients with confirmed ARC, considerations should be made for all renally cleared medications. Therapeutic drug monitoring and dose adjustment could be performed, when possible. Table 3 depicts suggested initial dosing of studied drugs in patients with ARC. For those medications where TDM is not routinely available and there is no sufficient evidence to guide dosage modification in ARC population, the use of the highest approved dose or most frequent administration regimen could be considered with close clinical monitoring. Furthermore, we suggest that consideration be made for alternative therapies that are not affected by altered renal function, such as those medications that are mainly metabolized rather than renally cleared (e.g., use of antiepileptic drugs that are eliminated via metabolism in place of levetiracetam). Finally, although attainment of therapeutic drug levels helps predict efficacy and safety, it is ultimately the patient’s clinical status and outcome that should be the anchor for all therapeutic decision making.

## Figures and Tables

**Table 1:** Summary of studies pertaining to ARC.

Author, Year	Study Type	Age (Years) Median (IQR)	Population	N	Sex (% Male)	Measured CrCl (mL/min)—Otherwise Specified	Intervention	Main Results
Barletta et al. [4], 2017	Retrospective observational	48 ± 19	ICU trauma patients where measured SCr available and SCr < 115 µmol/L	133	76	168 ± 65	ARCTIC (Augmented Renal Clearance in Trauma Intensive Care) Scoring system suggested	67% of patients identified to have ARC (CrCl > 130 mL/min) ARC risk factors identified: from multivariate analysis were age < 56 years, age = 56–75 years, SCr < 62 µmol/L, male sex ARCTIC score > 6 had sensitivity 84% and specificity 68%
Naeem et al. [5], 2017	Prospective observational	ARC: 37 ± 16 Non-ARC: 34 ± 14	ICU patients with SCr < 115 µmol/L	50	ARC: 70 Non-ARC: 60	ARC: 214 ± 46 Non-ARC: 112 ± 11	The effects of ARC on enoxaparin determined; Patients received enoxaparin 40 mg SC daily; Anti-Xa activity measured and compared among patients with and without ARC; measured 24 h CrCl	40% of patients identified to have ARC (CrCl > 130 mL/min) Anti-Xa activity did not differ in both groups at baseline and 4 h after administration ARC patients had significantly lower anti-Xa activity 12 and 24 h post enoxaparin administration; this implies short duration of action of enoxaparin in patients with ARC.
Udy et al. [6], 2017	Prospective observational (sub-study of BLING II RCT)	ARC: 52 (33–60) Non-ARC: 65 (55–73)	ICU patients with severe sepsis	254	ARC: 73 Non-ARC: 57	ARC: 165 (144–198) Non-ARC: 56 (27–83)	Conducted to determine the effect of ARC on patient outcome; patients randomized to receive beta lactam antibiotics (piperacillin/tazobactam, ticarcillin/clavulanic acid or meropenem) by intermittent or continuous infusion; measured 8 h CrCl	18% of patients identified to have ARC (CrCl > 130 mL/min); mainly younger, male and with less organ dysfunction No outcome differences (ICU-free days or mortality) between ARC and non-ARC No outcome differences were identified between continuous or intermittent infusion strategy
Udy et al. [7], 2017	Prospective observational	37 (24–49)	ICU (TBI patients with SCr < 120 µmol/L)	11	82	Median (day 1): 201 (76–289)	Measured 8-h CrCl, cardiac output and ANP were determined and correlated	ARC complicates TBI patients (Measured CrCl generally > 150 mL/min); mainly males and with young age CrCl was not significantly associated with changes in ANP or cardiac output
Barletta et al. [8], 2016	Retrospective observational	48 ± 18	ICU (trauma)	65	74	169 ± 70	Measured 12-h CrCl compared with CG method, CKD-EPI, and MDRD-4	69% of patients identified to have ARC (CrCl > 130 mL/min)—more common in younger patients and patients with SCr < 71 µmol/L Measured CrCl was significantly higher (*p* < 0.001) than all estimates of CrCl CG demonstrated lowest amount of bias (38 ± 56 mL/min) compared to CKD-EPI and MDRD-4
Chu et al. [9], 2016	Retrospective observational	Group A: 63 ± 15 Group B: 59 ± 14 Group C: 44 ± 16	Patients treated with vancomycin	148	66	Estimated by CG Group A: 54 ± 17 Group B: 106 ± 15 Group C: 188 ± 50	Vancomycin 1000 mg IV Q12H regimen given; vancomycin levels drawn pre 4th or 5th dose; levels compared across three groups: A (CrCl < 80), B (CrCl 80–129), C (CrCl ≥ 130; ARC)	Patients with ARC (Group C) had higher percentage of subtherapeutic vancomycin Vancomycin trough concentrations at steady state: Group A = 25 ± 10, Group B = 15 ± 8, Group C = 9 ± 5 mg/L Vancomycin trough concentration below 10 mcg/mL: Group A = 0%, Group B = 28%, Group C = 63%
Declercq et al. [10], 2016	Prospective observational	Abdominal Surgery: 63 (51–71) Trauma Surgery: 62 (46–75)	Non-critically ill surgery patients	232	Abdo. Surgery: 74 Trauma Surgery: 58	Abdominal Surgery: 109 (82–135) Trauma Surgery: 109 (85–142)	Aim to assess the prevalence of ARC in non-critically ill surgical patients; Measured 8-h CrCl	Abdominal surgery patients: 30% of patients identified to have ARC (CrCl > 130 mL/min) Trauma surgery patients: 35% of patients identified to have ARC (CrCl > 130 mL/min) The study identified presence of ARC in non-ICU surgery patients especially in younger male patients and undergoing trauma surgery
Hirai et al. [11], 2016	Retrospective observational	4.4 (range 1–15)	Pediatric ICU patients with normal renal function (Japan)	109	59	eGFR estimated by Schwartz formula 160 ( range 90–323) mL/min/1.73 m^2^	Vancomycin 40–60 mg/kg per day given in 2–4 divided doses; vancomycin clearance estimated	ARC defined as eGFR ≥ 160 mL/min/1.73 m^2^ Febrile neutropenia is an independent risk factor for ARC Age and eGFR significantly associated with vancomycin clearance (*p* < 0.0001)
Kawano et al. [12], 2016	Prospective observational	67 (53–77)	ICU (Japan)	111	56	Not reported	Measured 8-h CrCl	39% of patients identified to have ARC (CrCl > 130 mL/min/1.73 m^2^) Age < 63 years was identified as a risk factor for ARC
Roberts et al. [13], 2016	Prospective PK study	61 ± 17	Patients treated with levofloxacin	18	67	Estimated using CG 70 ± 67	Doses of levofloxacin 500 and 750 mg daily have been used; Monte-Carlo simulation conducted to determine PTA in ICU cohort compared to non ICU ones	For CrCl > 130 and CrCl > 200, levofloxacin 1000 mg Q24H provided the highest target attainment for *S. pneumoniae*, *P. aeruginosa* and *S. aureus*. For H. *influenza*, all levofloxacin doses/regimens analyzed were able to achieve >99% attainment
De Cock et al. [14], 2015	Prospective PK study	2.6 (range 0.08–15)	Pediatric ICU	50	60	Not reported	Population PK of amoxicillin/clavulanate in pediatric ICU population; Conventional dosing of 25–35 mg/kg every 6 h was tested.	In ARC patients; the best dosing is 25 mg/kg over 1 h every 4 h; it produced the best median target attainment by Monte-Carlo Simulation
De Waele et al. [15], 2015	Retrospective observational	62 (50–72)	ICU	1081	63	ARC: 178 (140–243) Non-ARC: 54 (32–82)	Measured 24-h CrCl and ARC risk factors determined	56% of patients identified to have ARC (CrCl > 130 mL/min): mainly younger and less likely to be treated with vasopressors Continuous ARC was present in 33% of patients ARC incidence 37 per 100 ICU patient-days
Dias et al. [16], 2015	Retrospective observational	Mean 42 (range 20–66)	ICU (TBI patients)	18	89	CG method 199 (Range 62–471)	Cerebrovascular pressure reactivity index (PRx) correlated with CrCl	A strong negative correlation between CG CrCl and PRX (*r* = −0.82, *p* = 0.001) have been reported This correlation suggests that reduction in cerebral autoregulation (i.e., after TBI) is associated with an increase in creatinine clearance.
Huttner et al. [17], 2015	Prospective observational	ARC: 41 ± 12 Non-ARC: 51:10	ICU with CrCl ≥ 60 mL/min	100	75	Estimated with CG ARC: 166 (145–200) Non-ARC: 103 (87–113)	They determined the influence of ARC on patient outcome; Standard dose antibiotic regimens given (imipenem/cilastatin 500 mg IV QID; meropenem 2 g IV TID; piperacillin/tazobactam 4.5 g IV TID; cefepime 2 g IV BID)	64% of patients identified to have ARC (CrCl > 130 mL/min); mainly younger ARC predicted undetectable antimicrobials concentrations but was not correlated with clinical failure
Morbitzer et al. [18], 2015	Retrospective observational	CN: 44 (29–52) TH/PI: 48 (40–62)	ICU (TBI)	27	63	Estimated using CG CN: 119 (91–166) TH/PI: 129 (100–156)	Vancomycin pharmacokinetics compared in patients with CN (T = 36–37 C), TH (T=33–34 C) or pentobarbital infusion	Vancomycin clearance was higher in controlled normothermic patients compared to predicted values based on population parameters
Ruiz et al. [19], 2015	Prospective observational	ARC: 39 ± 16 No ARC: 55 ± 19	ICU (patients with normal SCr)	360	ARC: 75 No ARC: 65	ARC: 173 ± 44 No ARC: 79 ± 30	Measured 24-h CrCl compared with 4 formulas to estimate CrCl/GFR (CG, Robert, MDRD and CKD-EPI methods)	33% of patients identified to have ARC (CrCl > 130 mL/min/1.73 m^2^): mainly trauma patients; younger Different formulas tended to overestimate CrCl for low eGFR values and underestimate CrCl for normal and high eGFRs Three factors suggested to identify ARC: Age ≤ 58 years, trauma and eGFR > 108.1 mL/min/1.73 m^2^ as calculated by CKD-EPI
Spadaro et al. [20], 2015	Retrospective observational	Group A: 63 ± 11 Group B: 71 ± 10	ICU	348	Group A: 73 Group B: 69	Group A: 106 ± 41 Group B: 37 ± 16	Vancomycin administration protocol based on measured 24 h CrCl and vancomycin serum measurements; levels compared between two groups: A (CrCl > 50) and B (CrCl ≤ 50) Vancomycin serum trough target: 15–25 mg/L	66% of patients had subtherapeutic vancomycin on second determination ARC and increased to 80% on third determination Patients who had a subtherapeutic vancomycin levels at the first determination had a significant correlation with in-hospital mortality (OR = 2, *p* = 0.003)
Steinke et al. [21], 2015	Prospective observational	66 (57–74)	ICU	100	61	73 (47–107)	Measured CrCl compared with estimated CrCl using serum cystatin C Hoek formula, CG, and CKD-EPI	16% of patients identified to have ARC (CrCl > 130 mL/min/1.73 m^2^): mainly trauma patients; TBI or SAH The Hoek formula’s precision was higher than CG and CKD-EPI Authors suggested more studies are needed to identify the rule of Hoek’s formula in drug dosing.
Adnan et al. [22], 2014	Prospective observational	34 (24–47)	ICU patients with SCr < 120 µmol/L)	49	76	ARC: 173 (141–223) Non-ARC: 91 (64–112)	Measured 24-h CrCl compared with CG method	39% of patients identified to have ARC (CrCl > 130 mL/min) CG method significantly underestimated CrCl in ARC patients
Akers et al. [23], 2014	Prospective observational	45 ± 19	ICU	13	62	Not reported	They determined the ability of the ARC score to predict piperacillin/tazobactam clearance; Piperacillin/tazobactam doses given were (3.375 g IV Q6H or 4.5 g Q6H)	ARC score had sensitivity 100%, specificity 71% in detecting enhanced clearance of piperacillin/tazobactam
Baptista et al. [24], 2014	Prospective observational	ARC: 49 ± 15 No ARC: 60 ± 18	ICU patients with normal SCr	54	ARC: 64 No ARC: 36	ARC Patients: 161 ± 28 Non-ARC Patients: 105 ± 16	Measured 8-h CrCl compared with CG method, CKD-EPI, and MDRD	56% of patients identified to have ARC (CrCl > 130 mL/min) All formulas underestimated 8h-CrCl for values >120 mL/min/1.73 m^2^ and a overestimated for values <120 mL/min/1.73 m^2^
Baptista et al. [25], 2014	Prospective observational (Group 1 data were retrospectively collected)	Group 1: 58 ± 16 Group 2: 60 ± 17	ICU	G 1: 79 G 2: 25	Group 1: 66 Group 2: 68	Group 1: 125 ± 67 mL/min/1.73 m^2^ Group 2: 121 ± 54 mL/min/1.73 m^2^	Continuous infusion Vancomycin dosing nomogram based on 8h measured CrCl was suggested Group 1: retrospective data Group 2: prospective assessment of the nomogram Target vancomycin level: 20–30 mg/L	36% and 40% of patients identified to have ARC in groups 1 and 2, respectively (CrCl > 130 mL/min/1.73 m^2^) Vancomycin clearance was significantly proportional to measured CrCl
Campassi et al. [26], 2014	Prospective observational	ARC: 48 ± 15 Non-ARC: 65 ± 17	ICU patients with SCr < 115 µmol/L	363	ARC: 48 Non-ARC: 47	ARC: 155 ± 33 Non-ARC: 78 ± 25	CrCl measured by 24 h urine collection; Vancomycin loading dose 15 mg/kg followed by continuous infusion 30 mg/kg/day was given Target trough 15–25 mg/L	28% of patients identified to have ARC (CrCl > 120 mL/min/1.73 m^2^); generally younger; more trauma and obstetric admissions; lower APACHE II scores 27% of patients who received vancomycin were identified to have ARC and had significantly lower vancomycin levels (*p* < 0.05) than non-ARC patients at 3 days; none of the ARC patients reached target trough despite being administered significantly higher vancomycin doses
Hites et al. [27], 2014	Prospective observational	61 (18–84)	Non-ICU obese (BMI ≥ 30 kg/m^2^) patients treated with antibiotics	56	50	107 (6–389.0)	They assessed the adequacy of serum concentrations of antimicrobials when given to obese individuals; Standard doses of antibiotics given (Cefepime 2 g TID, Piperacillin/tazobactam 4 g QID, Meropenem 1 g TID); Measured 24-h CrCl determined	Low levels of antimicrobials were detected following standard doses. Elevated CrCl was the only predictor of those low concentrations underlining the role of ARC in under-dosing obese individuals.
Udy et al. [28], 2014	Prospective observational	Mean 37 (95% CI 29 –44)	ICU patients with SCr < 120 µmol/L and age ≤ 60	20	60	Mean: 168 (95% CI 139–197)	Measured 24-h CrCl determined; various exogenous markers given to detect changes in nephron physiology	ARC involves increased glomerular filtration, renal tubular secretion of anions and renal tubular reabsorption using various exogenous markers, suggesting that ARC affects many components of the nephron physiology
Udy et al. [29], 2014	Prospective observational	Mean 54 (95% CI 53–56)	ICU patients with SCr < 120 µmol/L	281	63.3	Mean: 108 (95% CI 102–115)	Measured 8-h CrCl determined daily	65% of patients identified to have ARC (CrCl > 120 mL/min/1.73 m^2^); mainly younger, men, multi-trauma victims, and receiving mechanical ventilation Presence of ICU admission day 1 ARC predicted sustained ARC during ICU stay
Carlier et al. [30], 2013	Prospective observational	56 (48–67)	ICU	61	85	125 (93–175)	Meropenem or piperacillin/tazobactam were given as extended IV infusions; antibiotics concentrations measured; measured 24 h CrCl determined; Meropenem dose: an IV loading of 1 g over 30 min then 1 g Q8H as extended infusion over 3 h; Piperacillin/tazobactam dose: an IV loading of 4.5 g over 30 min then 4.5 g Q6H extended infusion over 3 h.	80% of patients identified to have ARC (CrCl > 130 mL/min) Elevated creatinine clearance is an independent predictor for not achieving meropenem and piperacillin/tazobactam target levels
Claus et al. [31], 2013	Prospective observational	ARC: 54 (44–61) Non-ARC: 66 (57–77)	ICU patients receiving antimicrobial therapies	128	ARC: 73 Non-ARC: 61	98 (57–164) mL/min/1.73 m^2^	Measured 8 h-CrCl determined; measuring the effect of ARC on antimicrobial therapy failure	52% of patients identified to have ARC (CrCl > 130 mL/min/1.73 m^2^); mainly younger and male 27% of ARC patients had therapeutic failure (poor clinical response to antimicrobial therapy), more often than non-ARC patients (13%) (*p* = 0.04)
Minkute et al. [32], 2013	Retrospective observational	ARC: 46 (21–66) Non-ARC: 54 (22–86)	Patients treated with vancomycin	36	80	Estimated CG ARC: 151 (131–324) Non-ARC: 103 (90–127)	Vancomycin level comparison between ARC and non-ARC groups	50% of patients identified to have ARC (CrCl > 130 mL/min, using CG) Vancomycin concentrations were lower in ARC group compared to Non-ARC Vancomycin doses up to 44 mg/kg/day were needed in the ARC group to achieve target trough levels.
Roberts and Lipman [33], 2013	PK study (analysis of Phase III trial data)	58 ± 15	ICU patients with pneumonia	31	93	Estimated by CG 137 ± 71	Population PK of doripenem in critically ill.	Doripenem clearance correlated with CrCl and was increased compared to non-ICU patients
Shimamoto et al. [34], 2013	Retrospective observational	Non-SIRS: 64 SIRS-2: 54 SIRS-3: 49 SIRS-4: 42	ICU (Septic patients on vancomycin)	105	66	Using CG No-SIRS: 121 ± 51 SIRS-2: 160 ± 65 SIRS-3: 195 ± 70 SIRS-4: 191 ± 77	Identified patients who had SIRS and categorized based on the number of SIRS criteria they had (non-SIRS, SIRS-2, 3 and 4); vancomycin CL and CrCL (CG) determined	In patients age < 50: higher SIRS score predicted higher vancomycin clearance In patients age > 50: higher SIRS does not reliably predict higher vancomycin clearance
Udy et al. [35], 2013	Prospective observational	42 ± 17	ICU (trauma, septic, SCr < 110 µmol/L)	71	63	Mean: 135 ± 52	They determined the prevalence and risk factors of ARC	58% of patients identified to have ARC; more in trauma patients; generally younger, males, with lower APACHE II , SOFA and higher cardiac index Three risk factors suggested to predict ARC: age < 50 years, trauma, and SOFA score ≤ 4 (ARC score)
Udy et al. [36], 2013	Prospective observational	51 ± 17	ICU patients with SCr < 121 µmol/L	110	64	Mean: 125 ± 45 mL/min/1.73 m^2^	Measured 8 h CrCl compared to estimated CrCl (CG and CKD-EPI)	CKD-EPI and CG underestimated CrCl in patients with ARC In patients with CKD-EPI eGFR = 60–119 mL/min/1.73 m^2^, 42% had ARC
Baptista et al. [37], 2012	Prospective observational	Non-ARC: 70 (52–79) ARC: 41 (32–56)	ICU septic patients on vancomycin	93	Non-ARC: 71 ARC: 79	Non-ARC: 70 (58–104) ARC: 159 (141–194)	The effect of ARC on vancomycin PK: ARC patients compared to non-ARC patients; measured 24 h CrCl Vancomycin dosing: A loading dose of 1000 mg if wt. < 70 kg or 1500 mg if wt. > 70 kg then 30 mg/kg/day continuous infusion	Serum vancomycin concentrations in ARC were significantly lower than control group for the 3 days of study
Grootaert et al. [38], 2012	Retrospective observational	59 (48–67)	ICU patients with measured CrCl > 120 mL/min (24-h method)	390	63	148 (132–172) mL/min/1.73 m^2^	Measured 24-h CrCl compared with CG method (CrCl) and 4-variable MDRD method (eGFR)	CG and MDRD underestimated measured CrCl in ARC patients
Udy et al. [39], 2012	Prospective observational	53 ± 21	ICU	48	71	134 ± 90	Measured 8 h CrCl; beta lactam antibiotic concentrations measured	For patients with trough less than MIC and less than 4× MIC: 82 and 72% had CrCl > 130 mL/min/1.73 m^2^, respectively A 25 mL/min/1.73 m^2^ increase in CrCl is associated with a 60% reduction in the probability of obtaining a trough concentration ≥ 4×MIC
Baptista et al. [40], 2011	Retrospective observational (post hoc analysis)	35 (25–51)	ICU patients with ARC	86	77	162 (145–190) mL/min/1.73 m^2^	Measured 8-h (Australia) or 24 h (Portugal) CrCl compared with CG, modified CG, 4-variable MDRD and 6-variable MDRD	All CrCl estimates underestimated measured CrCl CG estimates had the greatest sensitivity→identified 62% of cohort with ARC; lower sensitivity observed with 4-variable MDRD (47%) and 6-variable MDRD (29%) formulae
Minville et al. [41], 2011	Retrospective observational	NPT: 58 ± 17 PT: 42 ± 18	ICU	284	NPT: 63 PT: 75	NPT: 85 ± 5 PT: 131 ± 5 mL/min/1.73 m^2^	Measured 24-h CrCl; compared among patients with (NPT) and without polytrauma (PT)	37% of patients identified to have ARC (CrCl > 120 mL/min; significantly more trauma patients; younger; had lower SAPS II score; males Age and trauma independently correlated to CrCl
Spencer et al. [42], 2011	Prospective PK study	54 ± 14	Neuro ICU	12	42	96 ± 32 (estimated, method not reported)	Patients received levetiracetam 500 mg iv every 12 h; levetiracetam levels measured	Levetiracetam clearance was higher in neurocritical care population compared to healthy volunteers (drug clearance directly proportional to estimated CrCl *r*^2^ = 0.5, *p* = 0.01); Higher doses of levetiracetam are recommended in neurocritical care population
Goboova et al. [43], 2015	Case Report	16	ICU (Polytrauma and sepsis)	1	100	Method not reported Day 29: 138 Days 41–51: 340 mL/min/1.73 m^2^	Vancomycin initiated at doses of 1 g IV every 12 h then titrated up	A trauma patient who developed ARC later during hospital stay Vancomycin needed to be increased to up to 6 g per day to achieve therapeutic target trough level Vancomycin increased to 2 g Q12H, which produced troughs of 15 mg/L and 10 mg/L Increase in CrCl observed on day 46, vancomycin trough decreased to 5 mg/L, then dose increased to 2 g Q8H (67 mg/kg/day); trough: 19 mg/L
Abdul-Aziz et al. [44], 2014	Case Report	36	ICU (CNS infection)	1	100	234	Days 1–3: Flucloxacillin 2 g IV Q4H Days 4–16: Flucloxacillin 20 g/day via continuous infusion	A case report of an ICU patient with CNS infection Presence of ARC (Measured 8 h) lead to difficulty achieving therapeutic flucloxacillin concentration above MIC with conventional dosing (IV 2 g Q4H) A continuous infusion of high dose flucloxaciilin (20 g/day) was required to achieve clinical cure
Cook et al. [45], 2013	Case Report	22	Neuro ICU (TBI)	1	0	Method not reported 153	They described a case of ARC leading to subtherapeutic vancomycin and levetiracetam levels	A conventional vancomycin dose 750 mg IV Q12H (17.5 mg/kg/dose) yielded a trough level of 2.2 mg/L (subtherapeutic); dose had to be increased to 1.25 g iv Q8H (29 mg/kg/dose) to achieve a trough of 11 mg/L Levetiracetam dose was increased from 1000 mg IV BID to 1000 mg Q8H to maintain a trough within reference range (usual dose 0.5–1 g iv Q12H)
Lonsdale et al. [46], 2013	Case Report	44	Neuro ICU (SAH with ventriculitis)	1	100	375 mL/min/1.73 m^2^	They described a case of ARC leading to subtherapeutic vancomycin and meropenem levels	The patient required dose escalation of vancomycin up to 6 g/day (Vancomycin 63 mg/kg/day) in order to achieve therapeutic trough levels (usual dose 45 mg/kg/d for CNS infections) The patient required dose escalation of meropenem up to 2 g every 6 h in order to achieve therapeutic levels (usual dose 2 g iv Q8H for CNS infections)
Troger et al. [47], 2012	Case Reports	Pt 1: 37 Pt 2: 66	ICU patients with sepsis	2	100	Estimated with CG Pt 1: Initially: 138 Day 5: 276 Pt 2: Initially: 185 Later: 219	Described 2 cases of sepsis patients who required high doses of meropenem secondary to ARC Pt 1: meropenem 1 g IV Q8H then increased to meropenem 2 g IV Q4H Pt 2: meropenem 1 g Q8H then dose increased to1 g Q6H then to 2 g Q6H Meropenem trough target 4–10 mg/L	Pt 1: meropenem dose was increased to 2 g IV Q4H × 1 week, trough was still <4 mg/L but procalcitonin decreased and clinical improvement was observed Pt 2: meropenem dose was increased to 2 g Q6H, producing a trough concentration of 8.4 mg/L
Udy et al. [3], 2010	Case Series	Pt 1: 19 Pt 2: 41 Pt 3: 32	ICU Pt 1 TBI Pt 2 Surgery Pt 3 Burn	3	100	Pt 1: 224 Pt 2: 206 Pt 3: 151 Measured 8 h CrCl	Three case reports of patients with ARC Pt. 1: meropenem Pt 2: vancomycin + meropenem Pt 3: amikacin + ciprofloxacin	Pt 1: meropenem 1 g Q8H, increased to 2 g Q8H due to undetectable trough serum concentration; pt. still did not have detectable concentration Pt 2: vancomycin doses were increased up to 4 g (44 mg/kg) continuous infusion over 24-h for clinical cure; Meropenem increased to 1 g Q4H to reach target trough Pt 3: Amikacin 1 g daily was increased up to 1.2 g Q12H due to undetectable trough levels
Caro et al. [48], 2016 Abstract	Phase I PK study	Range 29–50	ICU patients with ARC (CrCl ≥ 180 mL/min estimated by CG)	5	40	Estimated using CG 282 (207–417)	Determined the PK of ceftolozane/tazobactam in patients with ARC	Study identified higher clearance of ceftolozane/tazobactam in ARC patients compared to non-ARC patients
Goboova et al. [49], 2016 Abstract	Retrospective observational	Mean 42 ± 14	Patients treated with gentamicin	204	78	Method not reported ARC Patients: 166 ± 28 mL/min/1.73 m^2^	Identification of the influence of ARC on gentamicin dosing	14% of patients identified to have ARC (CrCl > 130 mL/min/1.73 m^2^; 93% of ARC patients were under-dosed (low peak levels) with standard gentamicin regimens
Morbitzer et al. [50], 2016 Abstract	Prospective observational	63 (56–71)	Neuro ICU (Hemorrhagic stroke)	17	27	131 (108–216) mL/min/1.73 m^2^	Measured 8-h CrCl compared with CG method; vancomycin trough concentration determined	CG method underestimated CrCl in ARC patients Measured vancomycin trough was lower than predicted
Morimoto and Ishikura [51], 2016 Abstract	Prospective observational	Not reported	ICU (Japan)	33	Not reported	Not reported	CrCl measured (method not reported)	39% of patients identified to have ARC (CrCl > 130 mL/min/1.73 m^2^); mainly younger, less males, had lower APACHE II score and not septic
Dunning and Roberts [52], 2015 Abstract	A survey	N/A	N/A	123	N/A	N/A	A survey of 123 ICU physicians about antibiotic prescribing and renal function assessment	15% responded that they would consider modifying the dosage regimen of beta-lactams and vancomycin antimicrobials in patients with ARC This highlights the need for dosing guidance in patients with ARC.
Baptista et al. [53], 2014 Abstract	Retrospective observational	Not reported	ICU	477	Not reported	Not reported	CrCl measured by 8 h urine collection	33% of patients identified to have ARC (CrCl > 130 mL/min/1.73 m^2^) Urinary creatinine > 45 mg/mL and age < 65 are identified as best predictors of ARC (sensitivity 60%, specificity = 88%); specificity increased to 95% by adding BUN < 7 mmol/L
May et al. [54], 2014 Abstract	Prospective observational	Not reported	Neuro ICU (SAH)	20	Not reported	326 ± 135 mL/min/1.73 m^2^	Measured 24-h CrCl determined; Monte-Carlo Simulation for levetiracetam doses to achieve trough levels ≥ 6 mg/L	Estimated CrCl (method not reported) significantly underestimated CrCl Monte Carlo Simulation suggested that levetiracetam dosing poorly achieved target attainment unless TID was used (as opposed to standard regimen)
Vermis et al. [55], 2014 Abstract	Retrospective observational	Not reported	Patients with hematological malignancies	96	Not reported	CrCl estimated using CG ARC pts: 147 Non-ARC pts: 79	Aimed to determine the prevalence of ARC in a hematological population; Vancomycin continuous infusion: loading 15 mg/kg, maintenance 30 mg/kg given and titrated based on levels Vancomycin trough target: 20 mg/L	Therapeutic vancomycin trough targets were achieved with dose of 42 mg/kg/day in ARC patients (day 5 post initiation) and with 33 mg/kg/day in non-ARC (day 3 post initiation).
Weigel et al. [56], 2014 Abstract	Retrospective observational	55	ICU patients without renal replacement and receiving vancomycin infusion	287	69	ARC: MDRD eGFR > 130 Non ARC: MDRD eGFR < 130	A vancomycin loading dose of 20 mg/kg was given then adjusted by Therapeutic drug monitoring to target 20–25 mg/L; Vancomycin levels compared in patients with various degrees of eGFR using MDRD	Subtherapeutic vancomycin levels were more frequent in patients with MDRD > 130 mL/min (55%) i.e., ARC vs. patients with MDRD < 130 mL/min (29%) *p* < 0.001 Higher than conventional vancomycin dosing is recommended in patients with ARC
Neves et al. [57], 2013 Abstract	Prospective observational	55 ± 13	ICU	54	72	Mean: 138	Measured 8-h CrCl compared with CG method	ARC identified in 50% of samples Per subgroup analyses: CrCl > 120 mL/min/1.73 m^2^, CG underestimated measured CrCl; CrCl < 120 mL/min/1.73 m^2^, CG overestimated measured CrCl
Grootaert et al. [58], 2012 Abstract	Retrospective observational	66 (56–75)	ICU patients with measured CrCl available	1317	63	Not reported	Measured 24-h CrCl	16% of patients identified to have ARC (CrCl > 120 mL/min): mainly younger, taller, have lower BMI and had longer ICU stay 30% patients had at least 1 episode of ARC Relative duration of ARC per patient was 5 days in all pts, and 7 days in antimicrobial group
Drust et al. [59], 2011 Abstract	Retrospective observational	Not reported	ICU patients with CrCl > 120 mL/min	15	Not reported	>120	Meropenem plasma concentrations measured	27% of patients had appropriate meropenem plasma levels 67% of patients required higher doses of meropenem (up to 8 g/day) than recommended

ANP = atrial natriuretic peptide; APACHE II = Acute Physiology and Chronic Health Evaluation; ARC = Augmented Renal Clearance; CG = Cockcroft Gault equation; CKD-EPI = Chronic Kidney Disease Epidemiology; CN = controlled normothermia; CrCl = creatinine clearance; GFR = glomerular filtration rate; ICU = intensive care unit; IQR = interquartile range; MDRD = modification of diet in renal disease method; MIC = minimum inhibitory concentration; PK = pharmacokinetic; PT = patient; PTA = probability of target attainment; SAH = subarachnoid hemorrhage; SAPS II = Simplified Acute Physiology Score SCr = serum creatinine; SIRS = systemic inflammatory response syndrome; SOFA = sequential organ failure assessment score; TBI = traumatic brain injury; TH = therapeutic hypothermia. Age and CrCl reported in median (IQR) or mean ± SD.

**Table 2:** The ARC risk scoring systems.

	ARC Scoring System [23,35]	ARCTIC Scoring System [4]
Criteria	Age 50 or younger = 6 pts Trauma = 3 pts SOFA score ≤ 4 = 1 pt	SCr < 62 µmol/L = 3 pts Male sex = 2 pts Age <56 years = 4 pts Age: 56–75 years = 3 pts
Interpretation	0–6 points→low ARC risk 7–10 points→high ARC risk	>6 points→high ARC risk <6 points→low ARC risk
Sensitivity	100%	84%
Specificity	71%	68%

ARC = augmented renal clearance; ARCTIC = augmented renal clearance in trauma intensive Care (ARCTIC); SOFA = sequential organ failure assessment score; SCr = serum creatinine concentration; pt = point; pts = points.

**Table 3:** Suggested dosing recommendations of the studied drugs in adult ARC population.

Drug	Suggested Dosage	Suggestion Basis
Levetiracetam	Dose: 1000 mg iv Q8H	Monte-Carlo simulation [42]
Levofloxacin	Dose: 750–1000 mg iv Q24H	Monte-Carlo simulation [13] Levofloxacin 1000 mg Q24H provided the highest target attainment for *S. pneumoniae*, *P. aeruginosa* and *S. aureus*.
Meropenem	Dose: 2000 mg iv Q8H	A dose of 1 g iv Q8H reported to be inadequate [27,30] Doses up to 8 g per day might be required [59]
Piperacillin/Tazobactam	Dose: 4.5 mg iv Q6H Extended infusion of the dose over 4 h might be required	Monte-Carlo simulation [23] Subtherapeutic serum concentrations have been reported with intravenous doses of piperacillin/tazobactam 4.5 g every eight hours in 61% of the treated patients [17] Continuous infusion may not improve treatment outcome [6]
Vancomycin	Initial loading dose: 25–30 mg/kg followed by a maintenance dose of 45 mg/kg/day in divided doses Q8H or continuous infusion Therapeutic drug monitoring is recommended (target 10–20 mg/L or 15–20 mg/L depending on the indication)	Doses up to 44 mg/kg/day have been reported to be needed in patients with ARC [32] Dosage regimens lower than the suggested dosage have resulted in subtherapeutic levels [9,20,37] A dosing nomogram suggested by Baptista et al. allowed achievement of therapeutic vancomycin levels; average dose was ~47 mg/kg/day in patients with average weight 70 kg and measured CrCl 150 mL/min [25]

ARC = augmented renal clearance; CrCl = creatinine clearance; above recommended doses are based on observational studies and pharmacokinetics simulations. Above doses will need to be tested prospectively to assess its influence on patients’ outcome; for antimicrobials, different doses might be needed based on the susceptibility pattern of the microorganisms.
